# Heterodyne background-oriented schlieren for the measurement of thermoacoustic oscillations in flames

**DOI:** 10.1007/s00348-024-03890-1

**Published:** 2024-10-01

**Authors:** Sami Tasmany, Daniel Kaiser, Jakob Woisetschläger, Johannes Gürtler, Robert Kuschmierz, Jürgen Czarske

**Affiliations:** 1 Institute for Thermal Turbomachinery and Machine Dynamics, Graz University of Technology, Inffeldgasse 25/A, 8010 Graz, Austria; 2 Laboratory of Measurement and Sensor System Technique, Faculty of Electrical and Computer Engineering, Technische Universität Dresden, Helmholtzstrasse 18, 01062 Dresden, Germany

## Abstract

In aircraft engines, thermoacoustic oscillations in the combustion chamber contribute significantly to noise emissions, which, like all other emissions, must be drastically reduced. Thermoacoustic oscillations are not only a concern, they can also be beneficial in hydrogen combustion. This work demonstrates that thermoacoustic density oscillations with amplitudes at least an order of magnitude smaller than those resulting from density gradients in a turbulent flame can be detected using laser interferometric vibrometry. This improvement was made possible by heterodyning a carrier fringe system in background-oriented schlieren (BOS) recordings, which were subsequently analyzed using techniques commonly used for holographic interferometry. In comparison with other BOS evaluation techniques, the filtering of the individual frames in the Fourier domain offers a more efficient computational approach, as it allows for phase averaging of a high number of single recordings to reduce noise from turbulence. To address fringe pattern distortions and cross talk in the Fourier domain, which both have been observed by other authors, we propose background subtraction methods and an optimized background pattern. Additionally, the procedure provides a visualization tool for marking the high turbulence regions of heat release by the variations in fringe amplitude. Finally, the line-of-sight data are reconstructed using the inverse Abel transform, with the data calibrated by laser interferometric techniques, resulting in local values for density oscillations.

**Graphical abstract:**

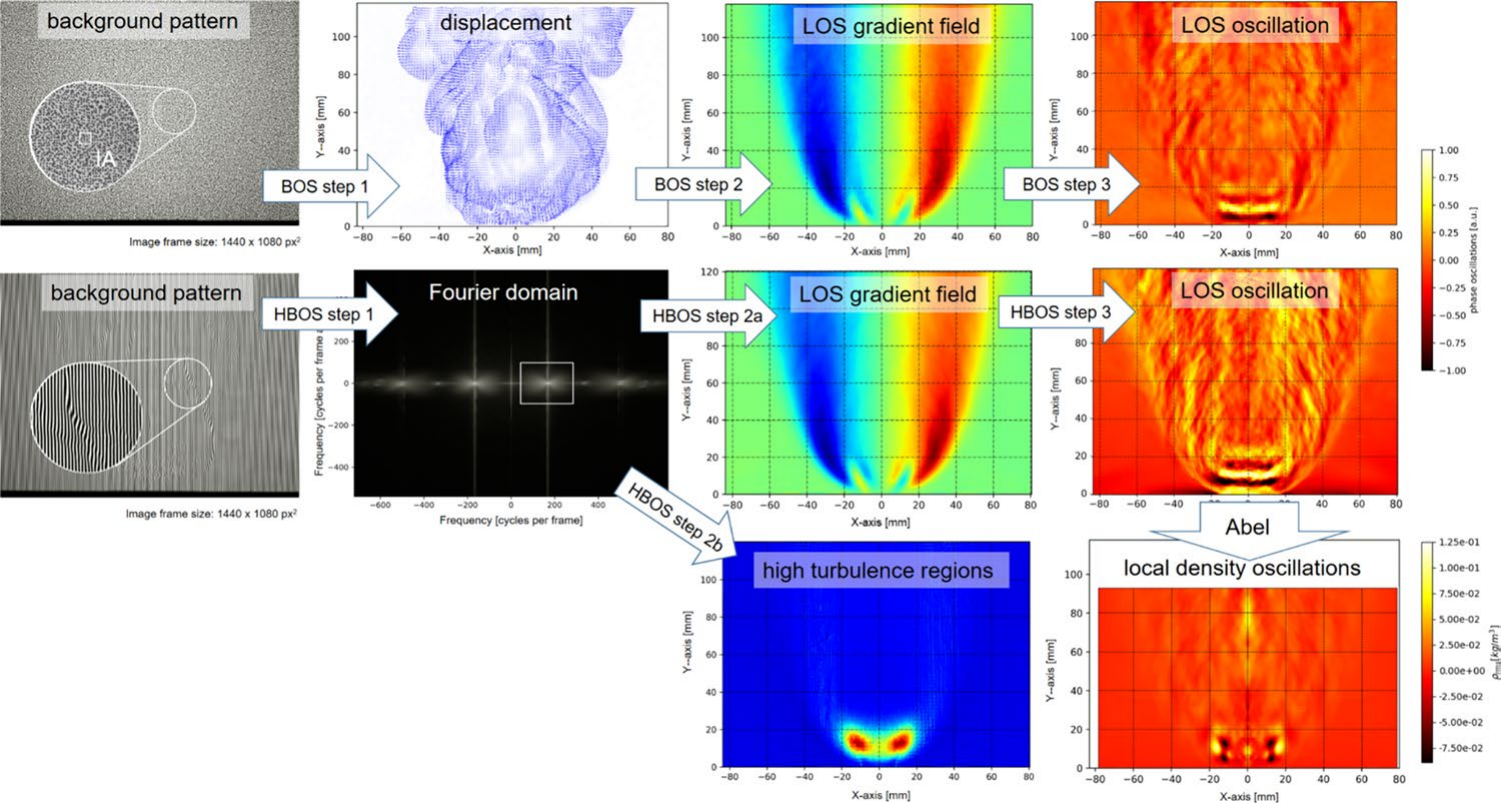

## Introduction

Thermoacoustic oscillation has troubled combustion engineers since the early days of the Apollo program (Oefelein and Yang 1993). Today, there is an increasing awareness regarding the necessity of dramatically reducing pollutant emissions and noise, becoming important to thermal turbomachinery used in ground-based gas turbines and aero-engines aiming toward climate-neutral aviation and sustainable fuels (Sacchi et al. 2023). Next-generation low-emission combustion modes burn more unsteadily, resulting in increased noise and instability from combustion due to the complex coupling between flow dynamics and acoustics, which is characteristic for the field of thermoacoustics (Dowling and Mahmoudi 2015). Thermoacoustics must also be considered for hydrogen combustion (Beita et al. 2021). Thermoacoustic forcing of premixed lean combustion of hydrogen can reduce *NO*_*x*_ emissions by increasing the combustion efficiency, the mixing rate, and the energy density in the flame (Paulitsch et al. 2023). Chemiluminescence is the most common tool in thermoacoustic research. However, there is an ongoing discussion on whether quantitative results on heat release oscillations can be obtained from this technique, based on the observation that mixture gradients and strain rate can influence the local emissions (Lauer and Sattelmayer 2010). Chemiluminescence is convenient to apply when optical access is granted, even though it is a line-of-sight (*LOS*) integration technique, meaning that multidirectional observation and tomographic reconstruction are needed for local data (Li and Ma 2015).

As an alternative, laser interferometric vibrometry (*LIV*) detects fluctuations in refractive index by scanning the combustion zone with a collimated laser beam (Greiffenhagen et al. 2019). The LOS fluctuations in refractive index detected by LIV can be related to density fluctuations by the Gladstone–Dale relation when variations in molar concentration (reactants vs. products) are limited, e.g., during lean combustion of hydrocarbons. Using conventional LIV, a total scanning time of more than 12 h may result, which is critical due to the instabilities of environmental conditions during measurement. Contrary to that, a camera-based LIV (*CLIV*) technique combines a LIV technology with a high-speed camera. CLIV technique is well suited for unsteady combustion and allows tracer-free velocimetry (Gürtler et al. 2021). Such a system captures the entire measurement volume simultaneously, similar to an array of several thousand single-beam LIVs, with the high-speed camera sampling at a recording rate of 200 kHz. To enable a carrier frequency of 50 kHz and frequency shifts in the range of ± 25 kHz in CLIV, a cascade of two acousto-optical modulators is needed. To ensure parallel light rays in the measurement volume, a high-quality and expensive telecentric lens system must be used, which limits the field of view (*FOV*) in the work published by Greiffenhagen et al. 2020 to about 50 × 50 mm^2^ with a resolution of 110 × 110 px, the latter due to the frame rate of 200 kHz of the high-speed camera. Although CLIV allows the study of transient processes with interferometric accuracy, the system is limited to a small area and unidirectional studies of thermoacoustic oscillations.

Another alternative is the background-oriented schlieren (*BOS*) technique (Gardner et al. 2019), a LOS integration technique recording lateral refractive index or density gradients in flames, offering a less limited FOV compared to CLIV. BOS is commonly accepted as a visualization tool in combustion (Weilenmann et al. 2018). Multidirectional observation of asymmetric flames with subsequent tomographic reconstruction is also possible by 3D-BOS (Grauer et al. 2018; Unterberger and Mohri 2022; Cai et al. 2022). As with CLIV, an estimation of the local (convection or advection) velocity in thermal flows is possible with BOS. The method of recording the advection of density structures through signal correlation is commonly referred to as density tagging velocimetry (Raffel et al. 2011; Gürtler et al. 2021). Neither BOS nor CLIV involve the addition of tracer particles to the flow for density tagging velocimetry, but both are LOS techniques requiring tomographic reconstruction to obtain local data. While LIV and CLIV are laser diagnostic tools that detect minute changes in the optical path length with interferometric accuracy and precision, BOS detects density gradients similar to a classic schlieren method. BOS, as proposed by Raffel (2015), is based on the deflection of speckle patterns and subsequent evaluation using a correlation technique for single interrogation regions, while other authors propose a fringe deflection technique (Perciante and Ferrari 2000; Blanco et al. 2016a, b; Vinnichenko et al. 2023). This approach borrows from a method known as (spatial) heterodyne holographic interferometry (Takeda 1990; Kreis 2005; Osten 1991), a fringe detection technique using a Fourier transform evaluation method and a spatial carrier frequency.

The purpose of this work is to explore the possibilities of such a heterodyne BOS (*HBOS*) for the detection of thermoacoustic oscillations in the refractive index field of an acoustically excited flame and to discuss its accuracy, with special emphasis on the high turbulence noise in combustion but small signals from periodic thermoacoustic oscillations. This work is based on the hypothesis that the sensitivity of BOS can be increased to the point where thermoacoustic fluctuations superimposed on the density distribution in a flame can be detected with sufficient accuracy. Calibration of these density fluctuations is then possible with a LIV system. The system should be capable of providing a large field of view and multidirectional observation, although the high turbulence of the flame necessitates the processing of a large number of images in a time- and computational-efficient manner.

This is possible since the field of view is not limited by a telecentric lens. Furthermore, since the instrumentation for HBOS is much cheaper compared to the high-speed camera of the CLIV, it is feasible to parallelize the acquisition process and perform a multidirectional observation with the least effort.

The test object is an open flame in the configuration already presented in Greiffenhagen et al. (2020), when LIV and CLIV were used to detect thermoacoustic oscillations in a swirl-stabilized methane flame operated at about 3.4 kW power, excited by a siren at 225 Hz. To benchmark the programming for HBOS, software packages on interferometric data evaluation algorithms (IDEA) subsumed under windows were used, accessible under http://optics.tugraz.at (Hipp et al. 2004). The IDEA software focuses on high-resolution digital fringe evaluation, including Fourier transform-based evaluation techniques, unwrapping techniques for regular and irregular fringe patterns, Abel inversion, and optical tomography.

## Methodology

### Background-oriented schlieren

**Fig. 1:**
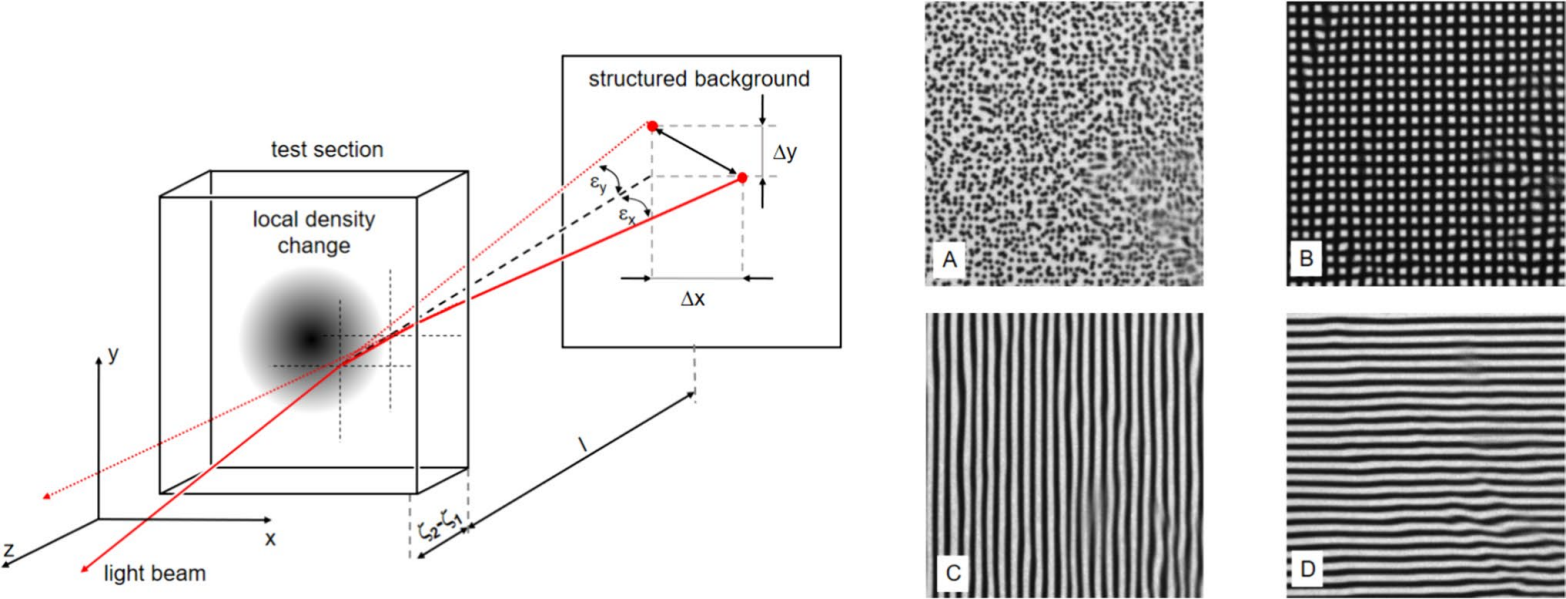
The left side of the figure discusses the flow visualization principle used by the background-oriented schlieren technique. When a pattern is viewed through a flow field with a density gradient, the background pattern appears to shift due to the refraction of light in the flow field. Different patterns that can be used as a background are plotted on the right side of the figure. **A** uses a speckle pattern as a background, **B** uses a grid, and **C** and **D** use fringe patterns. Two different types of distortion can be observed in these images. First, there is the deflection of the light rays at the gradients; second, there is a blurring of the image due to the high-frequency turbulence in the combustion (*n* is the refractive index, *x,y,z* the axis directions, ζ_1,2_ the integration limits, *l* the length between flame and background, and *ϵ*_*x,y*_ the deflection angle)

When a distant pattern is observed through a flow field with a density gradient, the background pattern shifts due to the refraction of light in the flow field. The LOS gradient of the refractive index is proportional to the observed displacement. The principle of a background-oriented schlieren technique is shown in Fig. 1. The displacement of the background pattern in the x-axis direction is given by Eq. 1, with *n* the refractive index, *x,y,z* the axis directions, *ζ*_*1,2*_ the integration limits, *l* the length between flame and background, and *ϵ*_*x*_ the deflection angle.

1 $$\Delta x=l\cdot \text{tan}{\varepsilon }_{x}=l {\int }_{{\zeta }_{1}}^{{\zeta }_{2}}\frac{1}{n}\frac{\partial n}{\partial x} \text{d}z= l \frac{\partial }{\partial x}{\int }_{{\zeta }_{1}}^{{\zeta }_{2}}\text{ln}n \text{d}z= l \frac{\partial }{\partial x} {(\text{ln} n)}_{\text{LOS}}$$

With BOS, the displacement of the background pattern can be determined by applying the same correlation algorithms as used for particle image velocimetry (*PIV*) if a gradient-free reference pattern is available (Raffel 2015). Local data can be derived from multicamera BOS projections using algorithms for tomographic reconstruction (Grauer et al. 2018; Unterberger and Mohri 2022; Cai et al. 2022). Raffel (2015) gives a detailed discussion of the BOS technique. A calibration can relate these local BOS data, which are still displacement data, to the absolute refractive index. In this study, we propose the use of a LIV or CLIV for calibration purposes. For a detailed analysis of the uncertainties of these interferometric methods, see Greiffenhagen et al. (2019; 2020).

In the lean methane flame used in this work, the variations in molar concentration between reactants versus products are small so that the linear Gladstone–Dale relation for the combustion gases can relate the refractive index to density fluctuations (Greiffenhagen et al. 2019; Gardiner et al. 1981). Density fluctuations measured in the combustion zone can then be linked to the fluctuations in heat release (Greiffenhagen et al. 2019).

**Fig. 2:**
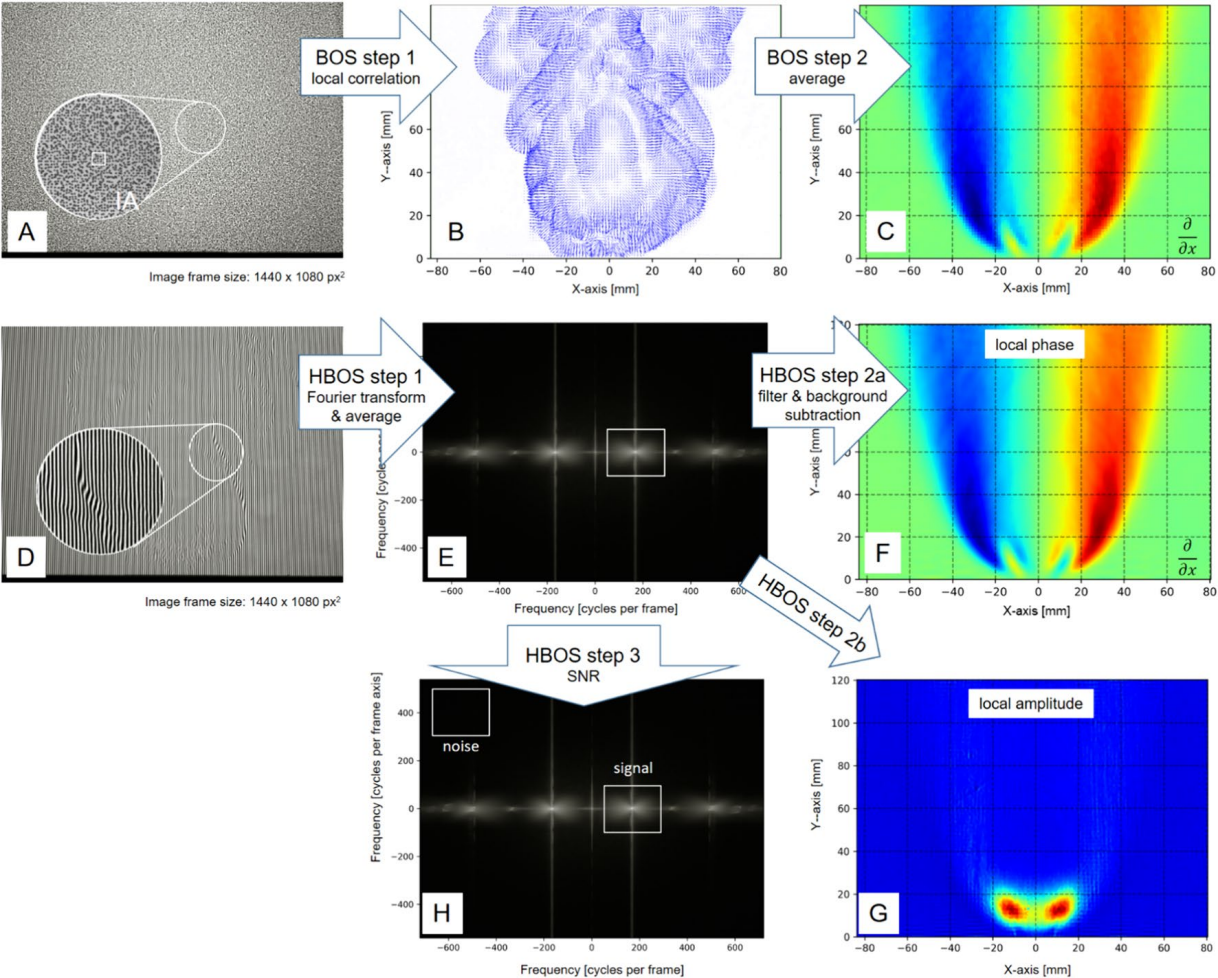
Image processing for BOS and HBOS. For BOS, the image frames (flame and gradient-free reference acquisition) are divided into interrogation areas (IA) by the PIV correlation algorithm **A**. For each IA, a Fourier transform-based correlation is performed. This process (BOS Step 1) results in a displacement field for a single instantaneous frame **B**. In BOS step 2, the data are validated and many frames can be averaged to obtain a gradient field, here in x-axis direction **C**. For HBOS, the individual frames **D** are first Fourier transformed (HBOS step 1) and then band-pass-filtered (indicated by the white rectangle in the frequency spectrum **E**). After an inverse Fourier transform in the next step and subtraction of background images—taken from gradient-free images—the phase distribution related to the gradient field perpendicular to the carrier fringe pattern (HBOS step 2a, **F**) and the fringe amplitude in the turbulent combustion regions are calculated (HBOS step 2b, **G**). The uncertainty in the phase distribution is related to the signal-to-noise ratio (SNR) in the windows used (HBOS step 3, **H**)

In Fig. 1, dotted (or speckled) background A is commonly used for BOS. The correlation algorithm for PIV divides the image frames, both the flame image and a gradient-free reference, into sub-frames called interrogation areas (*IA*). A correlation based on the Fourier transform is performed for each IA. The outcome of such a procedure is shown in Fig. 2, upper line, BOS step 1. It represents a displacement field for a single instantaneous recording. Obtaining representative data from a turbulent combustion requires up to 10,000 recordings. Fourier-based correlations must be performed for several thousand IA per recording, which makes this method computationally expensive. After data validation and averaging the displacements (Fig. 2, BOS step 2), the time-averaged LOS gradient field results. For maximum contrast in the single IA, the camera must focus on the background, not the object, with a sufficiently high number of single dots (Raffel 2015).

### Heterodyne background-oriented schlieren

Other authors proposed to use a background with parallel fringes or grid-like structures to detect the background pattern shift according to Eq. 1 (Perciante and Ferrari 2000; Blanco et al. 2016a, b; Vinnichenko et al. 2023). Superimposing a carrier frequency on a signal, in this case the pattern displacement, and then demodulating the signal is a well-known technique in holographic interferometry (Kreis 2004; Osten 1991). This spatial heterodyning and subsequent filtering in the spatial-frequency domain are a powerful tool for noise reduction, allowing phase shifts much smaller than a fringe spacing to be recorded (sub-fringe analysis). Like BOS, HBOS can reference a gradient-free fringe pattern to compensate for distortions in the optical path, e.g., by windows or uneven background patterns.

In the following, we discuss the intensity distribution *i*(*x,y*) of the fringe pattern in its sinusoidal base frequency. In Eq. 2*i*_0_(*x,y*) represents the low-frequency background intensity noise, such as that caused by uneven illumination, *a*(*x,y*) the fringe amplitude, *φ*_*c*_(*x,y*) the phase related to the carrier fringe system heterodyned, and *φ*_*d*_(*x,y*) the phase related to the displacement of the carrier fringe system. This displacement is the result of the deflection of light rays at the refractive index gradients.

2 $$i\left( {x,y} \right)\, = \,{i_0}\left( {x,y} \right)\, + \,a\left( {x,y} \right)cos[{\varphi_c}\left( {x,y} \right)\, + \,{\varphi_d}\left( {x,y} \right)]$$

*φ*_*d*_(*x,y*) is in a linear relationship to ∆*x* by 2π/*λ* with *λ* the carrier fringe period. Using Euler identity, Eq. 2 can be written in complex notation:

3 $$i\left( {x,y} \right)\, = \,{i_0}\left( {x,y} \right)\, + \,g\left( {x,y} \right)\, + \,{g^* }\left( {x,y} \right)$$

with

4 $$g\left(x,y\right)= \frac{1}{2} a\left(x,y\right){e}^{+j [{\varphi }_{c}\left(x,y\right)+ {\varphi }_{d}\left(x,y\right)]}$$

and ^∗^ denoting the complex conjugate. The initial step in the evaluation process is the Fourier transformation of the single image frames (Fig. 2, HBOS step 1). In the spatial-frequency domain, the carrier frequency and its frequency modulation have to be band-pass-filtered, as indicated by the white rectangle in Fig. 2. After an inverse Fourier transform in the next step (Fig. 2, HBOS steps 2*a* and 2*b*), the modulo 2*π* phase and the fringe amplitude distributions are calculated by:

5 $${\varphi }_{c}\left(x,y\right)+ {\varphi }_{d}\left(x,y\right)= \text{arctan}2 \frac{Im[g(x,y)]}{Re[g(x,y)]}$$

and

6 $$a\left(x,y\right)= 2 \sqrt{g(x,y){g}^{*}(x,y)}$$

After unwrapping the modulo 2π phase distribution, the [*φ*_*c*_(*x,y*) + *φ*_*d*_(*x,y*)] phase distribution is obtained. Background subtraction, which uses gradient-free phase and amplitude fields, must be performed for both the phase and the fringe amplitude distributions obtained from Eqs. 5 and 6. This is necessary to subtract the phase distribution *φ*_*c*_(*x,y*) and compensate for local perturbations of the carrier fringes. The result is the phase distribution *φ*_*d*_(*x,y*), which is linearly related to the displacement field *∆x*(*x,y*). The uncertainty in phase is related to the signal-to-noise ratio (*SNR*) in the window used for band-pass filtering (Rathien 1995; Takeda 1990; Freischlad and Koliopoulos 1990; Whalen 1971). The spatial resolution in the final gradient map is reduced according to the Nyquist–Shannon theorem and the window’s width. Figure 2F plots the time-averaged gradient field perpendicular to the carrier fringe pattern.

The fringe amplitude *a*(*x,y*) is subject to variation due to the blurring and defocusing effects of strong gradients and high-frequency turbulence regions, which are primarily generated in the combustion zone and occur at time scales faster than the camera’s exposure time. Due to the LOS nature of BOS and HBOS, the high-frequency and small-scale turbulence is underestimated in fringe amplitude blurring (Mayrhofer and Woisetschläger 2001). Thus, the reduction of fringe amplitude is only a qualitative measure for the high turbulence and combustion regions in the flame. Figure 2G plots the fringe amplitude reduction in the turbulent combustion regions.

### Laser interferometric vibrometry

Another laser optical technique, LIV, was used to calibrate the results of HBOS for the detection of thermoacoustic oscillations. Commercially available laser vibrometers can detect optical path length oscillations caused by refractive index oscillations with sub-nanometer resolution for the 225 Hz thermoacoustic oscillations discussed here. Using an acoustic-optical modulator, these instruments record the change in optical path length instead of amplitude, providing a calibration factor k [mm s^−1^ V^−1^] that relates output voltage and velocity. As with BOS and HBOS, the refractive index oscillations can be related to the density oscillations using the Gladstone–Dale relation for the combustion gases, with the Gladstone–Dale number *G*, and the density *ρ*:

7 $$\frac{\text{d}}{\text{d}t} \underset{2\zeta }{\overset{{\phantom{a}}}{\int }}n \text{d}z= G \frac{d}{dt} \underset{2\zeta }{\overset{{\phantom{a}}}{\int }}\rho \text{d}z$$

where *f* is the oscillation frequency. The time derivative of the LOS oscillations in Eq. 7 is related to the LOS oscillations by 2π*f*. For LIV, the laser beam passes through the flame twice along the beam path *ζ*. The refractive index fluctuations detected are caused by pressure fluctuations and heat release fluctuations. When only the combustion zone is considered, the contribution of heat release to the density is dominant. Greiffenhagen et al. (2019, 2020) provide a detailed analysis of this technique, and an application of LIV to the study of acoustically perturbed premixed flames is given by Li and Ma (2015).

### Optical tomography

Optical tomography methods, including ray tracing and self-learning algorithms, are currently used to reconstruct the local distribution of the refractive index from BOS and CLIV data (Grauer et al. 2018; Unterberger and Mohri 2022; Cai et al. 2022; Rothkamm et al. 2021). To explore the potential of HBOS for detecting thermoacoustic oscillations in the refractive index field of an acoustically excited flame, the test object is a swirl-stabilized open flame operated at low power in a configuration already presented by Greiffenhagen et al. (2020). For this flame, an inverse Abel transformation can be used to reconstruct the local refractive index field in the radial direction *n(r)*, since the cylindrical symmetry requirement in the heat release fluctuations was met within a few percent for all operating points (cmp. Greiffenhagen et al. 2020).

8 $$n\left(r\right)= -\frac{1}{\pi }\int \frac{\text{d}{n}_{LOS}}{\text{d}x}\frac{1}{\sqrt{{{x}^{2}-r}^{2}} } \text{d}x$$

Various numerical methods for Abel inversion are discussed by Pretzler et al. (1992). Note that LOS gradients can be used directly to solve Eq. 8, as suggested by Pretzler et al. (1993).

## Experimental setup and numerical evaluation

### Experimental setup

#### Burner and feeding lines

**Fig. 3:**
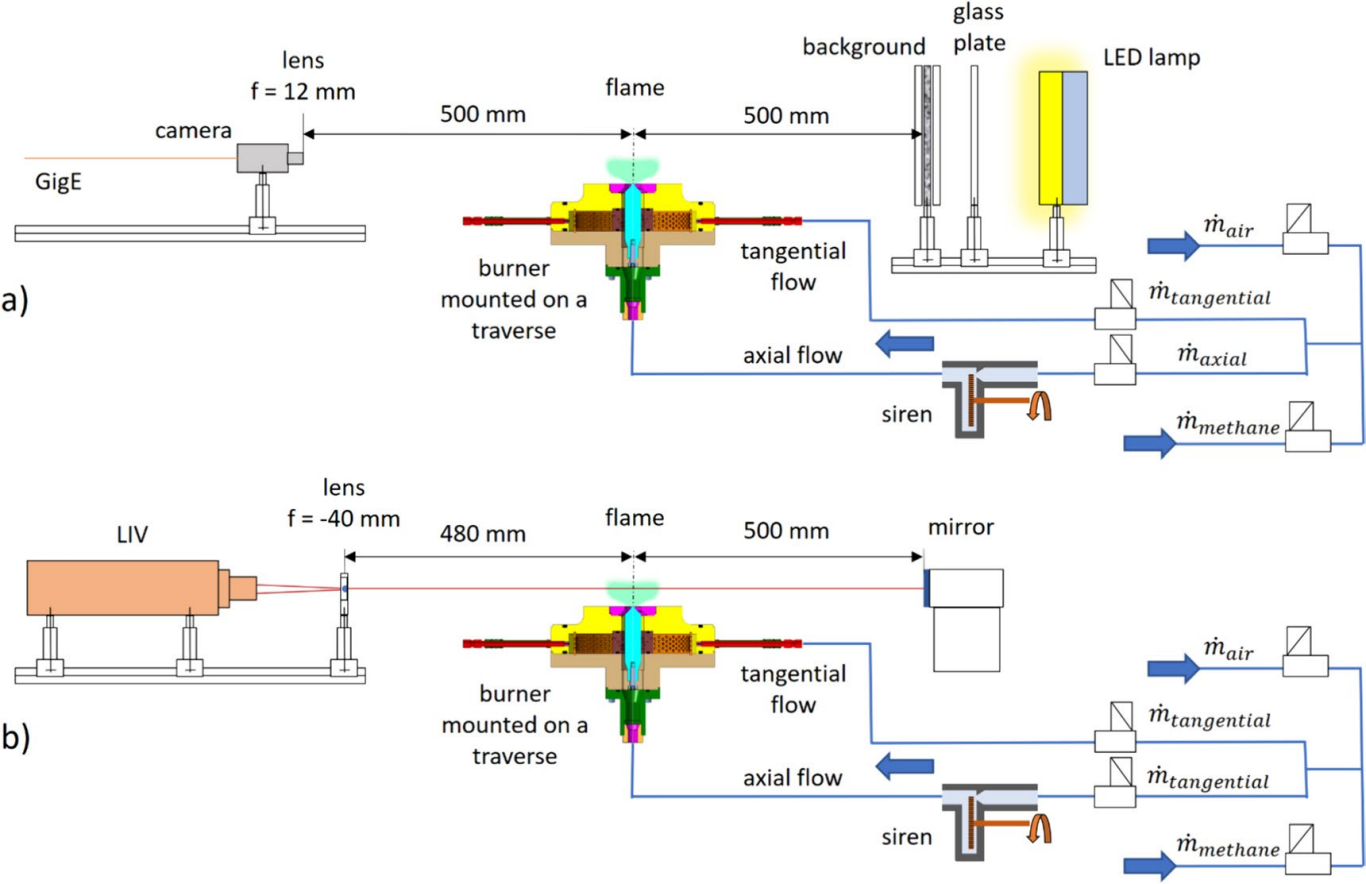
In section **a**, the experimental configuration for BOS and HBOS imaging procedures is outlined. The background pattern is glued onto a glass plate and secured by a top glass plate. A third glass plate is positioned at the back to shield the heat radiation emitted by the LED lamp. Section **b** explains the experimental setup for LIV measurement. The burner is moved along three axes to scan the flame with the LIV system

A schematic of the instrumentation is shown in Fig. 3. For the proof of concept in this work, we used a flame with a power of about 3.4 kW, while in future work the use in full-size combustors is planned, as previously shown (Peterleithner et al. 2016). The laboratory receives air and methane flows from two different storage lines. The methane pressure is regulated to approximately 8.00 bar and the air pressure is regulated to approximately 6.25 bar before entering the system shown in Fig. 3. The methane and air pressures are first measured separately and then combined; ELFLOW Select flow meters (Bronkhorst High-Tech B.V., Ruurlo, The Netherlands) are used on the system for all mass flow measurements, while mass flow control is performed using a set of needle valves. After mixing the methane and air, the feed lines deliver the resulting mixture to the burner via the axial and tangential flow paths. Again, the mixture delivered by the tangential line is then transported to the burner through many radially positioned inlets after passing through a plenum. Instead, a single axially positioned inlet carries the flow mixture supplied in the axial line to the burner after it has been metered and passed through a siren.

The siren is a device equipped with an opening that is periodically blocked by the teeth of a rotating gear (Giuliani et al. 2020). This device makes it possible to introduce pressure fluctuations at 225 Hz in the axial supply line. In addition, an infrared photointerrupter sensor GP1A51HR (SHARP, Osaka, Japan) generates a trigger signal from the siren. As a last step, the axial and tangential flows merge and pass through the burner nozzle where the mixture can be ignited by an external spark, resulting in an open swirl-stabilized lean flame. Table 1 shows the mass flow conditions at the burner, the equivalence ratio was 0.95, the swirl number 0.53. All conditions were similar to the unconfined flame published by Greiffenhagen et al. (2020).

**Table 1:** Mass flow rates in burner’s supply lines

${\dot{m}}_{\text{air},\text{ tot}}$	1.320 g/s
${\dot{m}}_{\text{methane}, \text{tot}}$	0.074 g/s
$\frac{{\dot{m}}_{\text{tangential}}}{{\dot{m}}_{\text{axial}}}$	0.803

#### Background-oriented schlieren and heterodyne background-oriented schlieren

BOS and HBOS used the same setup presented in Fig. 3a with a camera and backlit background. A GigE monochromatic camera with a 12 mm focal length lens (C23-1224-5 M-P, Basler AG, Ahrensburg, Germany) was placed at a distance of 500 mm from the burner, facing the flame itself. The camera used was an acA1440-73gm (Basler AG, Ahrensburg, Germany) equipped with a CMOS sensor (Sony IMX273) with a resolution of 1440 × 1080 px and a pixel size of 3.45 µm. In object space, one pixel is approximately 0.1 mm long in this setup. The camera was set to a gain of 110. A 2 mm glass plate was placed on the opposite side of the camera. A given pattern was printed on a diffuse photographic paper and then glued to the side of the plate that was facing the flame. To ensure that there is no unwanted movement of the printed pattern during the measurement, another 2 mm glass plate was clamped on top of the photographic paper. These two glass plates and the printed pattern are referred to as the background. To illuminate the background, five battery-powered LED lamps (800–4500 lm Li-Ion 5200 mAh 14.8 V) of 50 W each were used. To shield the background plate from the heat radiated by the lamps and to prevent unwanted thermal expansion, a 6 mm thick glass plate was placed between the lamps and the pattern plate.

An aperture of F 5.6 was set on the camera's lens in order to capture useful images with a desired exposure time of 500 µs. The camera was focused on the background pattern for both BOS and HBOS. Figure 3 shows the relevant distances used in the system. The background was chosen after several preliminary tests with different grids and background patterns, which are discussed in Kaiser (2023). The BOS background pattern was plotted on a diffusing paper (RTL49 A3, Altiel Limited, UK) using the speckle pattern algorithm from the Python library (Python 3.9, Python Software Foundation, Wilmington, DE, USA). The parameters for this Python standard random speckle pattern were as follows: a speckle size of 0.75 mm, a position randomness of 2, and a grid spacing of approximately 1.5 times the speckle size. Four different background patterns were used for the HBOS technique. A vertical fringe pattern with 1 mm fringe thickness and fringe period and a horizontal fringe pattern with 1 mm fringe thickness and fringe period, both obtained from the speckle pattern Python library. In addition, a 1 mm grid pattern obtained from an author modification of the speckle pattern algorithm, and a modified grid pattern obtained by filtering the 1 mm grid pattern in the frequency domain to reduce the spectral diagonal components of the image signal.

The choice of this fringe period results from a trade-off analysis performed in Kaiser (2023). While a thicker fringe pattern leads to a lower spatial resolution, a thinner fringe pattern leads to a loss of signal when displacement and defocus are strong. This consideration holds for the current experimental setup and scale; another optimal fringe period may be more appropriate for other experimental setups.

### Laser interferometric vibrometry

The system used for the LIV measurements consisted of an OFV-503 sensor head (Polytech GmbH, Waldbronn, Germany) and an OFV-5000 vibrometer controller (Decoder VD-06, Polytech GmbH, Waldbronn, Germany) with a 20 kHz low-pass filter and a sensitivity of 2 mm s^−1^ V^−1^. A lens with a focal length of -40 mm was located 480 mm from the flame axis to obtain a collimated beam of approximately 1.5 mm in diameter. The collimated beam passes through the flame zone and is then reflected by a mirror placed on the opposite side of the burner from the sensor head. This mirror is located 500 mm from the flame axis. Figure 3b shows a schematic of the system. To derive the RMS voltage at 225 Hz, the signal from the LIV was sampled for 60 s at a sample rate of 16,384 Hz and evaluated at a resolution of 1 Hz.

#### Numerical evaluation of the background pattern

The BOS speckled background patterns were evaluated using the PIV algorithms of the Dynamic Studio software (Dantec Dynamics 7.5.19, Skovlunde, Denmark). This involved a cross-correlation of image pairs of the background patterns taken with and without the flame is performed within a 24 × 24px IA without overlap. HBOS fringe pattern evaluation was performed by the authors' Python routines, which were benchmarked against the Interferometric Data Evaluation Algorithms (IDEA) software package, to verify the correct usage of the algorithms. The Python routines follow the heterodyne procedure described in Kreis (2005), with a Fourier transform, a band-pass filtering of the carrier signal and its modulation, next a back-transform of the filtered signal, and finally an unwrapping of the modulo 2π phase. The Fourier transform and filtering were performed by dft module from the Python open cv library, unwrapping of the modulo 2π phase distribution by the unwrap_phase module from scikit-image library.

In both procedures, images are processed to obtain background pattern displacements due to flame-induced refractive index gradients. For both the BOS and HBOS methods, time-averaged and phase-averaged fields are calculated. Phase averaging means that, based on a periodic trigger signal derived from the monofrequency stimulation of the siren, each image is initially assigned a phase of the oscillation cycle. All of the captured images that correspond to a particular phase of the oscillation cycle are then averaged together. Here, the oscillation period was divided into eight phase steps. The time-averaged images were taken without a trigger in free-run mode. Thermoacoustic oscillations can then be calculated from the difference between time-averaged and phase-averaged data.

To tomographically reconstruct the data from the LOS data, the Python Abel.direct module of the PyAbel library was employed. All Python routines used are enclosed in a repository (Tasmany and Woisetschläger 2024).

## Results and discussion

### Comparison of background-oriented schlieren and heterodyne background-oriented schlieren techniques

**Fig. 4:**
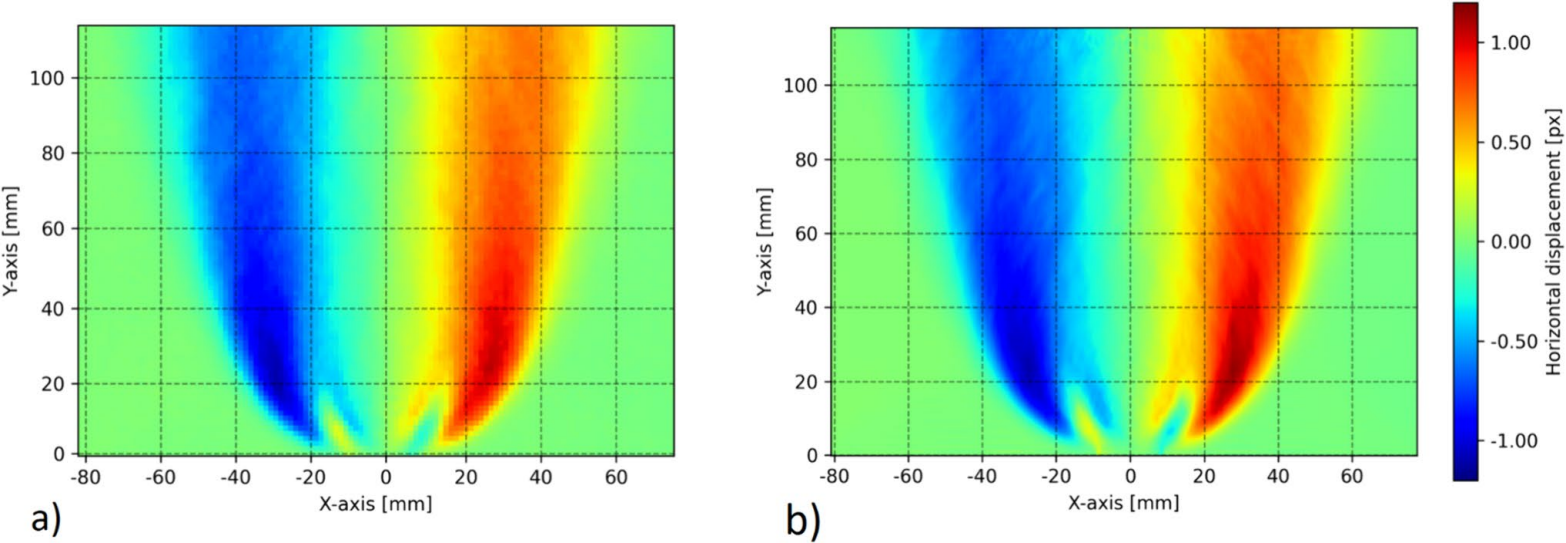
Comparison of horizontal displacements derived from **a** BOS with speckled background and an IA size of 24 × 24 px, and **b** HBOS with vertical fringes, with 8.6 px per period equals 2π. Each plot is a time average of 1000 recordings. One recording means one frame with the flame and one frame of the background without the flame

Figure 4 shows a comparison of the horizontal displacement fields in pixels obtained with the BOS and HBOS methods, respectively, for time-averaged data sets of 1000 instantaneous recordings. One recording means one frame with the flame and one frame of the background without the flame. Both methods give similar results, and the magnitudes of the displacements are in the same range for both methods. HBOS spatial resolution is not subject to the reliable cross-correlation constraint and can therefore achieve much higher resolution than standard BOS. However, it is important to note that the frequency domain filtering operation, which acts as a band-pass filter, introduces a resolution limit. This limit is much smaller than the one introduced by cross-correlation. In order to obtain reliable cross-correlation signals in the BOS method using the speckled background, it is necessary to keep the IA size above a certain value in order to record enough speckle pairs in the IA to obtain a low noise correlation signal. For the configuration under consideration, the resulting IA had a size of 24 × 24 px, resulting in an uncertainty of ± 0.11 px in the recording of the speckle pattern displacement with the IA size chosen (uncertainty estimate by Dynamic Studio from cross-correlation peak ratio). Here, the field was measured without overlap between the single IAs, as this is only an interpolation between individual IAs and does not alter the smoothing effect of IA correlations.

### Discussion of heterodyne background-oriented schlieren technique

**Fig. 5:**
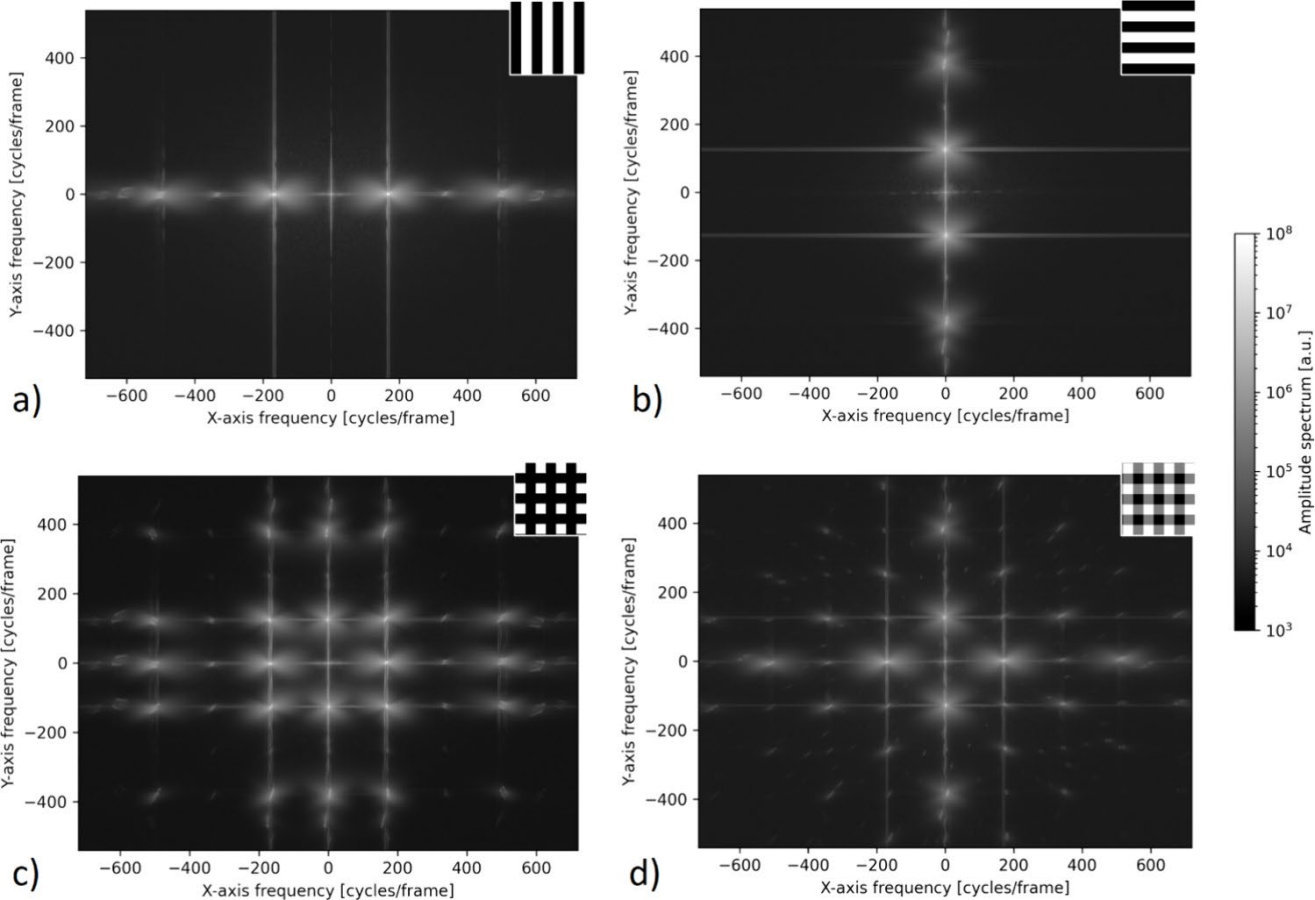
Averaged frequency spectra of HBOS fringe patterns recorded with flame. In the sections **a** and **b** the averaged spectra of the vertical and horizontal carrier fringes are compared, respectively. Section **c** shows the frequency spectrum of a standard grid, while section **d** shows the frequency spectrum of a grid with reduced intensity in diagonal direction to minimize cross talk between signals from different directions (optimized grid). 10,000 spectra were averaged in each case. The image size is 1440 × 1080 px. In the upper right corner of each spectrum, a section of the fringe pattern is shown

Figure 5 shows the final Fourier spectra (logarithmic scale) averaged over 10,000 spectra from the single images taken during the experimental campaign with flame. Due to the rectangular intensity distribution of the fringe patterns, the transformed signals also contain higher order harmonics. A band-pass filter between 63 cycles per frame up to 272 cycles per frame along the abscissa and a height of ± 70 cycles per frame was used to evaluate the vertical fringe patterns (window size: 210 × 140 cycles per frame). For the horizontal fringe patterns, a band-pass filter ranging from 49 cycles per frame to 205 cycles per frame and a width of ± 94 cycles per frame was applied along the ordinate (window size: 188 × 157 cycles per frame). Then the spatial resolution in the final displacement field is about 3.5 px in the horizontal and vertical directions, according to the Nyquist–Shannon theorem and the given frame size of 1440 × 1080 px. The same number of images was acquired for the gradient-free background as for the flame, with the same band-pass filter applied. The corresponding Fourier spectra were then averaged and processed. Averaging the images first and then Fourier transforming them would actually speed up the processing, but due to the high turbulence it would also reduce the fringe contrast and SNR in the averaged images.

As described in Sect. 2.1, after an inverse Fourier transform of the filtered frequency spectrum and unwrapping of the modulo 2π phase, the phase of the gradient-free background images was subtracted to obtain the local phase *φ*_*d*_(*x,y*), which is linearly related to the displacement field *∆x*(*x,y*) by the carrier fringe period *λ* of about 8.6 px.

9 $$\frac{2\pi }{\lambda }= \frac{{\varphi }_{d}(x,y)}{\Delta x(x,y)}$$

The fringe period can vary over the image due to image distortions, which must be taken into account when calculating the displacement field. Here, the local variations in the carrier frequency were less than 0.5% and subsequently ignored.

The SNR was calculated for the 400 × 400 px flame section using a band-pass filter ranging from 18 cycles per frame to 76 cycles per frame and a height of ± 20 cycles per frame, for both axes. The background noise was taken from the upper left corner in the frequency domain from an area of the same size (as indicated in Fig. 2H). Three different approaches were used to estimate the displacement uncertainty, all of which are listed in Table 2. When a grid is used as a background, the cross talk between the fundamental frequencies becomes visible in Fig. 5c. To reduce the fundamental frequency at the 45° angle and minimize the cross talk, an optimized grid was designed with reduced intensity in the diagonal axis. However, there is a trade-off between minimal cross talk and a reduction in SNR due to the reduced fringe contrast at the corners (Table 2). Although Table 2 clearly indicates that separate vertical and horizontal grids have the highest SNR, it is highly desirable to record both directions simultaneously, particularly for transient processes. A comparison between the standard grid and the optimized grid revealed that the increase in SNR for the standard grid is primarily due to the additional signal from cross talk present in the filter applied.

**Table 2:** Uncertainties in HBOS estimated from the SNR in the flame section for 10,000 spectra averaged

	SNR	Uncertainty [px]					
		A		B		C	
*Flame*							
vertical fringes, horizontal gradient	11 476	±	0.019	±	0.018	±	0.014
horizontal fringes, vertical gradient	12 441	±	0.018	±	0.017	±	0.014
standard grid, horizontal gradient	5 541	±	0.027	±	0.026	±	0.018
standard grid, vertical gradient	5 450	±	0.027	±	0.026	±	0.018
optimized grid, horizontal gradient	4 958	±	0.029	±	0.027	±	0.019
optimized grid, vertical gradient	4 723	±	0.029	±	0.028	±	0.019
*Background*							
vertical fringes, horizontal gradient	13 497	±	0.017	±	0.017	±	0.014
horizontal fringes, vertical gradient	13 523	±	0.017	±	0.017	±	0.014
standard grid, horizontal gradient	6 263	±	0.025	±	0.024	±	0.018
standard grid, vertical gradient	6 034	±	0.026	±	0.025	±	0.018
optimized grid, horizontal gradient	5 401	±	0.027	±	0.026	±	0.019
optimized grid, vertical gradient	5 265	±	0.028	±	0.027	±	0.019

In A the uncertainty is estimated according to Whalen (1971) using Gaussian distributions for signal and noise; in B the uncertainty is derived from uncertainty propagation (Rathjen 1995); and C uses a statistical approach from the image SNR (Takeda 1990). All methods estimate uncertainty similarly, though the image SNR slightly underestimates it

### Visualization of the thermoacoustic oscillation

**Fig. 6:**
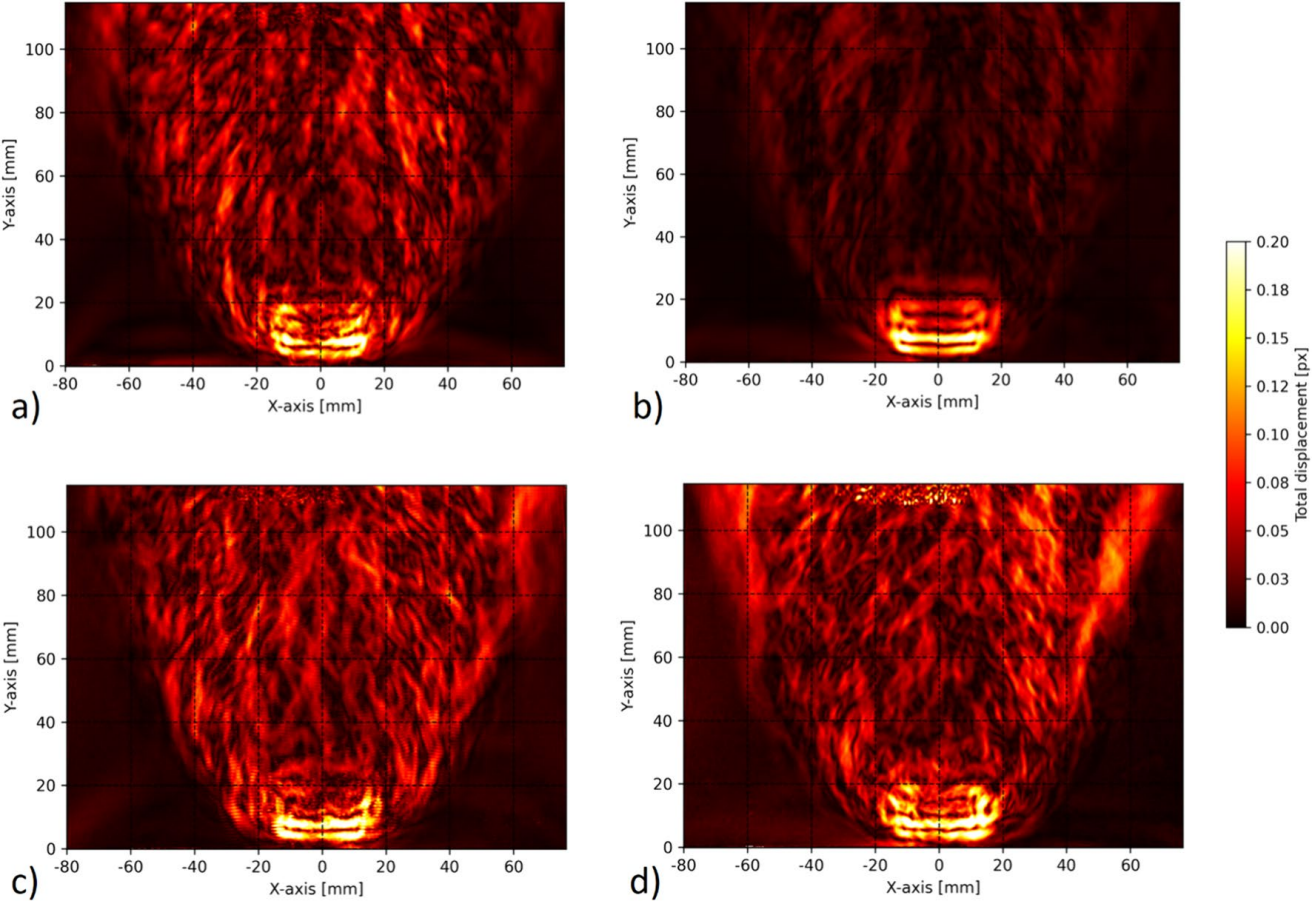
Displacement fields calculated from the vectorial difference between the phase-averaged and time-averaged fields for one phase step. Sections **a** and **b** show the displacements recorded by the vertical and horizontal fringe patterns for 1000 and 10,000 recordings, respectively. The comparison between **a** and **b** shows that large ensembles must be averaged to reduce the turbulent noise of a flame. Section **c** shows the result for the standard grid, while section **d** shows the result for the optimized grid, both with an average of 1000 recordings. The spurious fringe pattern in section **c** is caused by cross talk between diffraction orders

**Fig. 7:**
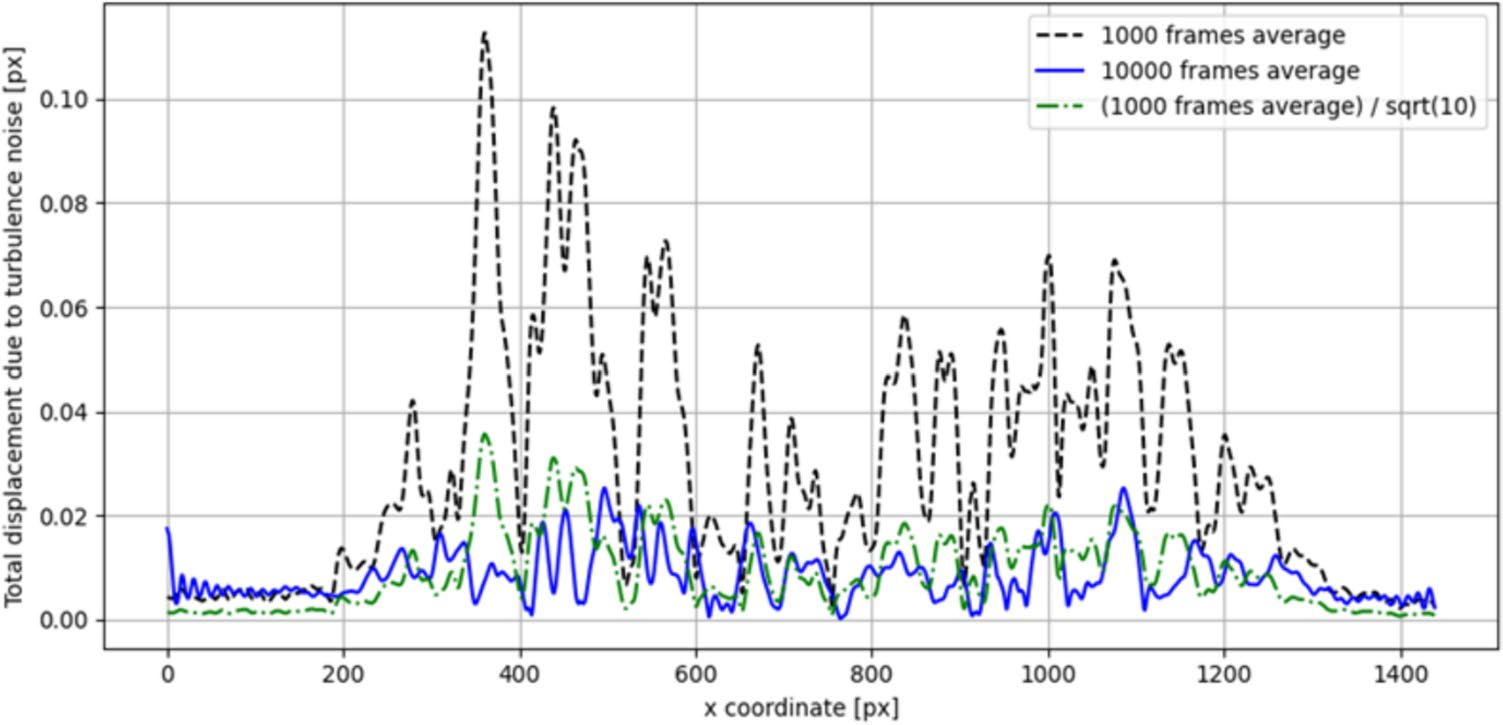
Horizontal line values extracted from the total displacement vectorial-difference fields in Fig. 6 at a height of 60 mm above the burner. This region is dominated by turbulent convection. An average over 1000 frames (dashed line) and an average over 10,000 frames (*solid line*) are considered for the cases plotted in Fig. 6a and b. For comparison, the average over 1000 frames is divided by the square root of 10 and plotted as dot-dashed line. It can be seen how the turbulent noise decreases with the number of samples, making the thermoacoustic oscillation more clearly recognizable in Fig. 6b

Figure 6 plots the magnitude of the displacement calculated from the vectorial difference between the phase-averaged and time-averaged fields for one phase step. The plots in Figs. 6a and b show the displacements recorded by the vertical and horizontal fringe patterns for 1000 and 10,000 recordings, respectively. The figures illustrate the impact of high turbulence within a flame superimposed on the low-amplitude thermoacoustic oscillations of interest, as well as the necessity to average over a large number of recordings. It is worth noting that when considering data obtained from a single fringe direction, the result is not complete to characterize the total displacement. In fact, it is necessary to know the gradient in both directions, so both fringe directions are needed, especially for neural network-based multidirectional and volumetric reconstructions. The tenfold increase in the number of samples used for the averaging process between Fig. 6a and b has resulted in a reduction of Gaussian distributed turbulence noise by a factor of the square root of 10. As shown in Fig. 7, increasing the number of samples by a factor of 10 reduces the perceived noise in the averaging by a factor close to the square root of 10. This is statistically justified by the fact that the standard deviation of an average value decreases with the square root of the number of samples for independent and identically distributed samples. It is important to note that evaluation of 10,000 recordings is feasible with the Python program written by the authors, but it becomes a time-consuming procedure when using BOS evaluation with the interrogation area-based Dynamic Studio software. Figure 6c and d compares the result for the standard grid and the optimized grid, both with an average of 1000 recordings. The spurious fringe pattern in Fig. 6c at coordinates (−15, 5) is caused by the cross talk between diffraction orders. In the open flame studied and under atmospheric conditions, the refractive index is so close to unity that the plots in Fig. 6 represent the oscillations in the LOS refractive index or density gradients (cmp. Eq. 1). In high-pressure combustors, this difference corresponds to the logarithm of the LOS density gradient ratios. In the case of a grid/optimized grid pattern, the horizontal and vertical displacements were recorded simultaneously, while in the case of fringe patterns, it is necessary to record one direction first and then the other after changing the background pattern. In this case, the displacements of the two directions come from two different points in time, but are always triggered by the siren. As previously outlined by Greiffenhagen et al. (2019), the displacement and gradients in the flame region are significant for fluctuations in heat release, while the region above the flame is dominated by turbulent convection of exhaust gases.

In addition to the displacement field, HBOS also allows the detection of variations in fringe amplitude *a*(*x,y*). These variations are the result of blurring and defocusing effects caused by strong gradients and high-frequency turbulence regions. These are primarily generated in the combustion zone and occur at time scales faster than the camera’s exposure time.

**Fig. 8:**
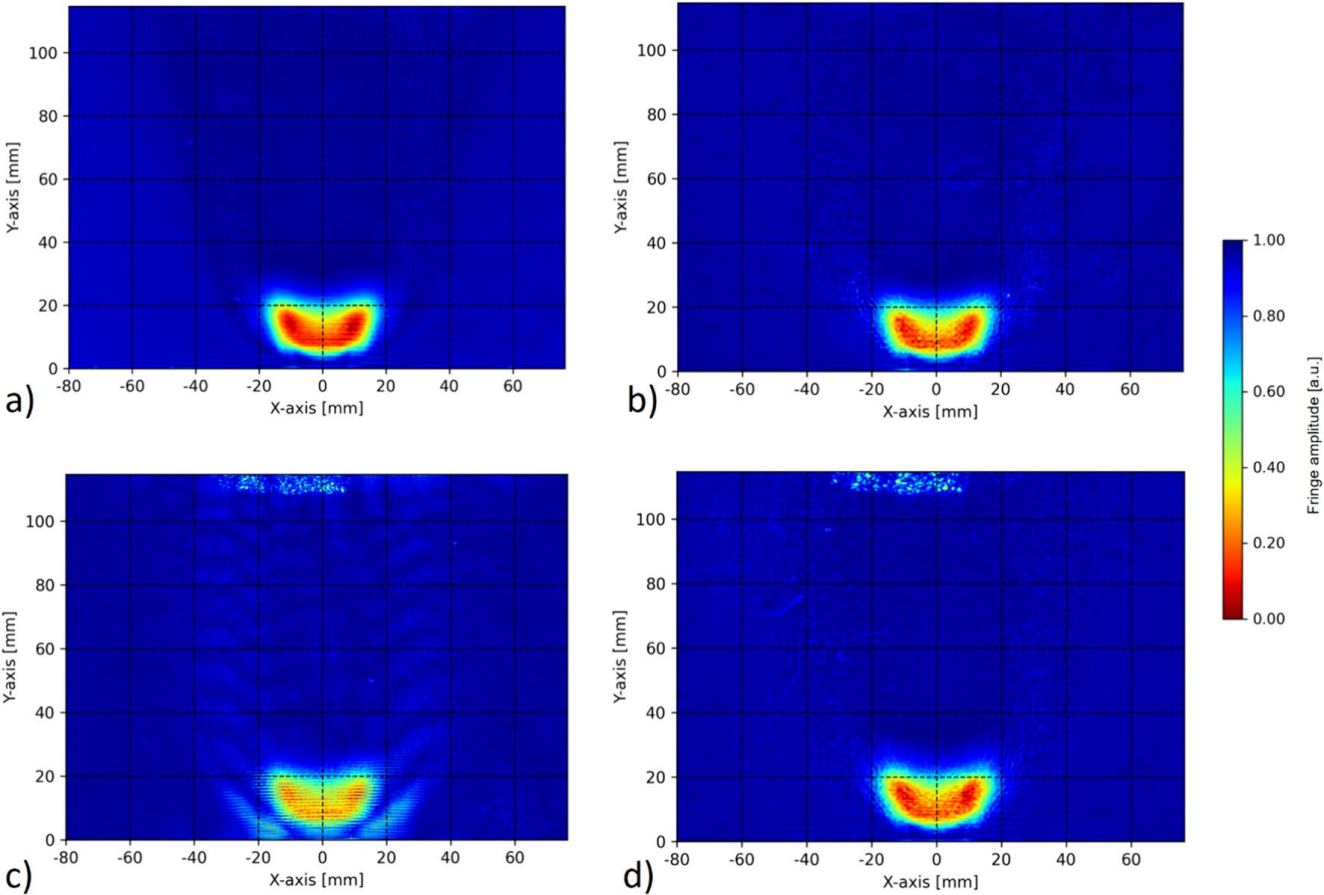
Normalized fringe amplitudes from HBOS evaluation of different fringe patterns. Section **a** shows the sum of the fringe amplitudes in the vertical and horizontal directions, while section **b** presents the results for the optimized grid, both averaged over 10,000 recordings. Sections **c** and **d** compare the standard grid with the optimized grid for 1000 recordings averaged, but with a significantly larger window used for the inverse Fourier transform. The cross talk with the 45° diffraction pattern can be seen in section **c**

The normalized fringe amplitude fields obtained by the HBOS method are shown in Fig. 8. Figure 8a plots the normalized fringe amplitudes for the sum of the horizontal and vertical fringe patterns, while Fig. 8b shows the amplitude for the optimized grid, both averaged over 10,000 recordings. Figure 8a provides a clear result due to the higher signal-to-noise ratio (SNR) (see Table 1). Please note that both the horizontal and vertical fringes must be recorded in separate test runs, while Fig. 8b is from the same test run. Figure 8c and d compare the standard grid, each for an average on 1000 recordings. Figure 8c shows the standard grid with a filter size of 200 × 200 px, starting at the same x coordinate as with the filter given in section 4.2. This demonstrates the influence of cross talk among the first orders in the standard grid.

It is important to note that in the combustion zone, these plots are similar in shape to the OH* emission plots shown in Greiffenhagen et al. (2020), which mark the location of heat release. While only qualitative in nature, this is an important feature of HBOS and is related to the detection of high-frequency turbulence in the combustion zone. Therefore, HBOS provides a second means for identifying heat release fluctuations in the combustion zone.

### Tomographic reconstruction

**Fig. 9:**
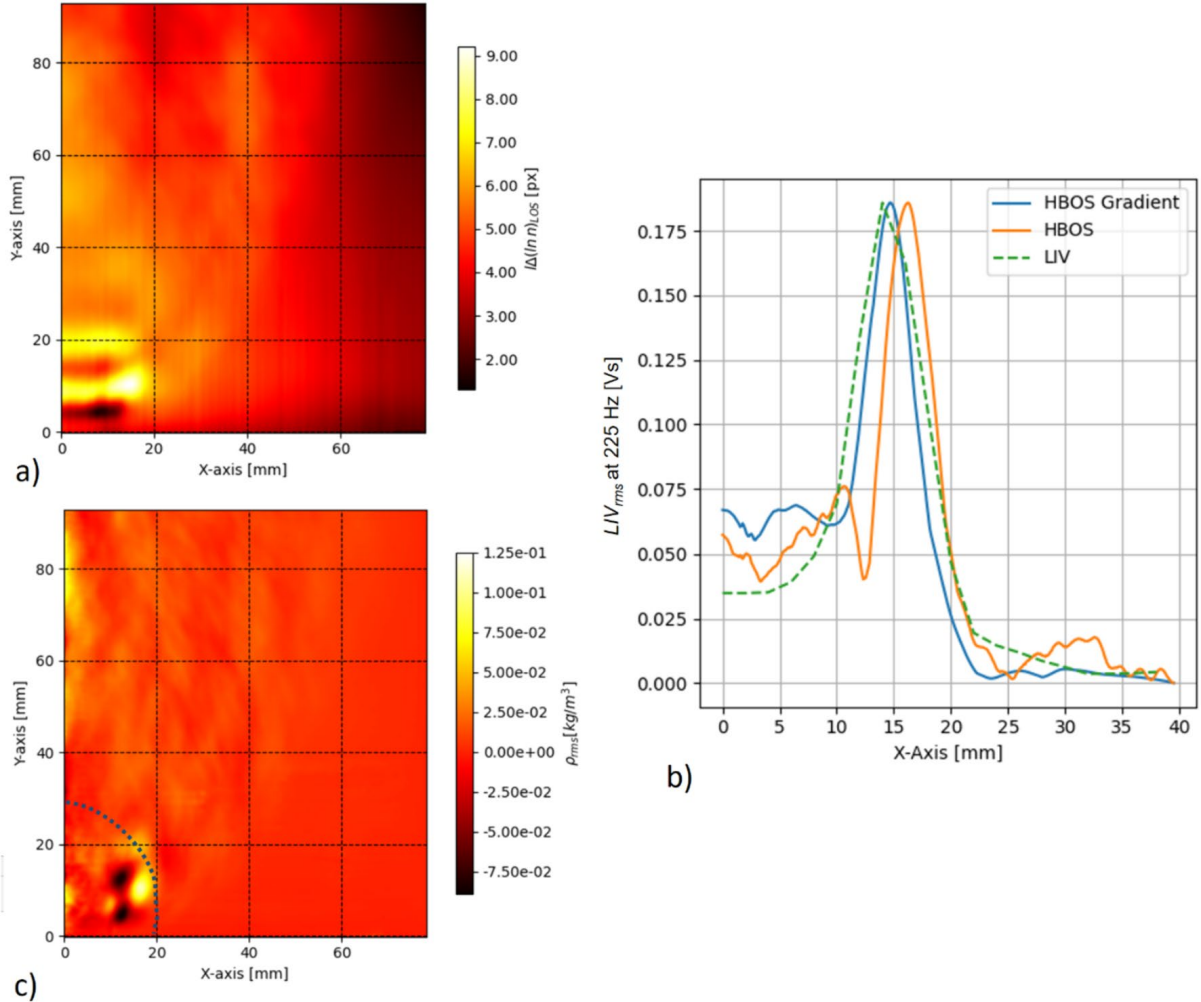
Tomographic reconstruction of the local refractive index oscillations at 225 Hz using an inverse Abel transformation. Section **a** plots the difference between the time-averaged and phase-averaged LOS data for a single phase step. These LOS data were derived by iteratively summing the gradient data. Section **b** discusses the difference between the LOS data recorded by LIV and the LOS data integrated from the tomographic reconstruction for the HBOS data (HBOS) or the HBOS gradient data (HBOS gradient). Section **c** plots the result of the tomographic reconstruction and calibration with LIV data for a half-plane cross section along the flame axis. It shows oscillations in density. The dotted circle indicates the FOV of the CLIV system used in Greiffenhagen et al. (2020) to discuss the same unconfined swirl-stabilized methane flame as discussed here. The oscillation phase step shown in section **c** corresponds to the 250° phase step presented in Greiffenhagen et al. (2020, Fig. 8)

In this case, an inverse Abel transformation was applied to tomographically reconstruct the local oscillations in refractive index from the local difference of time-averaged and phase-averaged HBOS data. Prior to applying the inverse Abel transformation, the LOS refractive index gradient data had to be transformed into LOS refractive index data. For this purpose, the horizontal gradients were first summed, and then these distributions were iteratively corrected with the sums of the vertical gradients. Figure 9a illustrates the difference between the LOS time-averaged and LOS phase-averaged refractive index data for a single time step. As mentioned above, the refractive index in the open flame studied under atmospheric conditions is so close to unity that the plot in Fig. 9a represents the oscillations in the LOS refractive index or density (see Greiffenhagen et al. 2020). Alternatively, the inverse Abel transformation allows the direct use of the gradient field for tomographic reconstruction (see Eq. 8). In both cases, the data had to be symmetrized by averaging the right and left half-plane data. After inversion, an exponential function was applied to these local data fields (time-average and phase-average data) to extract the local refractive index data. The difference between time-averaged and phase-averaged fields yielded the local oscillations in refractive index.

In the next step, the local data were integrated along the projection direction to be calibrated with the LOS data recorded by the LIV system for a plane 10 mm above the nozzle. Thus, the data integrated along the line of sight from the reconstructed field were scaled according to the LIV LOS data values. In this way, the calibration procedure relied on the integral data of the high-precision LIV reference. Figure 9b compares the LOS LIV data (dashed line) with the LOS data derived from either the local HBOS refractive data (green line) or the local HBOS refractive index gradient data (yellow line). It is apparent that the noise introduced by the summation process used to produce the refractive index LOS data from the gradient data and the subsequent derivation by the inverse Abel transformation affects the quality of the reconstruction. Finally, the local fluctuations in refractive index are calibrated to density fluctuations using Eq. 7. The Gladstone–Dale number was derived according to Gardiner et al. (1981) using the gaseq software (Morely 2005) to obtain the molar concentrations for reactants and products in the combustion zone. For lean combustion of hydrocarbons, the variation of the Gladstone–Dale number between products and reactants is less than 0.5%, so we used an average value of 2.45 10^–5^ m^3^ kg^−1^.

The result of the tomographic reconstruction and calibration with LIV data is shown in Fig. 9c for a half-plane cross section along the flame axis. A portion of the field is circled by a dotted line to indicate the FOV of the high-speed CLIV data discussed in Greiffenhagen et al. (2020). The oscillation step shown in Fig. 9c corresponds to the 250° phase step presented in Greiffenhagen et al. (2020, Fig. 8).

It has to be mentioned that the total density field in this cylindrically symmetric flame acts as a diffusing lens and distorts the image, in the case studied here uniformly and only slightly (image distortions less than 0.5%; cmp. Section 4.2). In the case of asymmetric flames, where a multidirectional observation is necessary, ray tracing must be performed in order to compensate for local distortions in the field. One potential avenue for achieving this is by applying deep learning programs that are similar to those used in adaptive optics (Sun et al. 2024).

Finally, it is necessary to discuss which systematic uncertainties arise due to the special features of thermoacoustic oscillations. A swirl-stabilized flame is characterized by turbulence and strain. Steinberg and Driscoll (2009) discuss how a counter-rotating vortex pair affects an initially undisturbed flame front. By curling the surface, strain is induced in the curved flame front in front of and behind the vortex pair. This results in the formation of a spatially separated zone of high expansion at the downstream (upper) vortex and compression below the upstream vortex. This leads to locally increased or decreased heat release and a corresponding change in density, as illustrated in Fig. 9c at coordinates (x,y) = (15,10), but also in local variations for molecular concentrations of reactants or products. In all the techniques discussed here, the refractive index must be related to the density by the Gladstone–Dale number, which varies slightly between reactants and products in the lean combustion of hydrocarbons. In this unconfined flame, cold ambient air can also mix with the hot exhaust products, leading to variations in the Gladstone–Dale number of up to ± 9% (cmp. Greiffenhagen et al. 2020).

## Summary and conclusions

In this study, the proposed HBOS, which combines a background-oriented schlieren method with principles of digital interferometry and phase averaging of a large number of images, allows to measure possible density fluctuations caused by thermoacoustic oscillations whose amplitude is an order of magnitude smaller than the turbulent density fluctuation in these flows. HBOS makes this possible by heterodyning carrier fringe systems, which were then analyzed using analysis methods from digital interferometry with sub-fringe accuracy, and consecutive phase averaging of up to 10,000 images from a highly turbulent swirl-stabilized flame. The fast filtering of the single frames in the Fourier domain provided a computationally less expensive method compared to other BOS evaluation methods. In the same amount of time, the HBOS code elaborated eight directories each containing 1000 images. Dantec Dynamic Studio takes even more than that for elaborating only one directory of 1000 images, on the same machine, and for having comparable results. The use of background subtraction and an optimized grid helped to reduce image distortions and cross talk in the Fourier domain. The data were calibrated by laser interferometric methods and, after inverse Abel transform, quantitative local data of density oscillations were derived and discussed in comparison with previous publications on thermoacoustic oscillations using the CLIV method. Additionally, the variations in fringe amplitude provide a visualization tool for marking the location of high turbulence regions. This is an important feature of HBOS and can be related to the detection of high-frequency turbulence rather than refractive index fluctuations, providing a second tool for visualizing heat release fluctuations.

In conclusion, this approach allows the simultaneous multidirectional reliable detection of thermoacoustic oscillations. Future tasks in this project will focus on multidirectional detection and density tagging velocimetry, employing machine learning tools to address the limited number of projections and the potential restrictions in real-sized test rigs.

## Data Availability

Sequence data that support the findings of this study have been deposited as required by the funding organizations at 10.3217/7k9f0-7wb03. The Python routines utilized in this project are also available for download from this repository (Tasmany and Woisetschläger 2024). This repository comes with a CC-BY4 legal code.
